# Dietary Intake of Endocrine Disrupting Substances Presents in Environment and Their Impact on Thyroid Function

**DOI:** 10.3390/nu13030867

**Published:** 2021-03-06

**Authors:** Aneta Sokal, Sara Jarmakiewicz-Czaja, Jacek Tabarkiewicz, Rafał Filip

**Affiliations:** 1 Institute of Health Sciences, Medical College of Rzeszow University, 35-959 Rzeszow, Poland; sjczaja@ur.edu.pl; 2 Institute of Medicine, Medical College of Rzeszow University, 35-959 Rzeszow, Poland; jacek.tabarkiewicz@gmail.com (J.T.); r.s.filip@wp.pl (R.F.); 3 Department of Gastroenterology with IBD Unit, Clinical Hospital No. 2, 35-301 Rzeszow, Poland

**Keywords:** endocrine-disrupting chemical, endocrine signaling, diet

## Abstract

According to the available data, environmental pollution is a serious problem all over the world. Between 2015 and 2016, pollution was responsible for approximately nine million deaths worldwide. They also include endocrine disrupting chemicals (EDCs) that can interfere with the functioning of the thyroid gland. They are characterized by high persistence in the environment. These substances can enter the body through the gastrointestinal tract, respiratory system, as well as contact with the skin and overcome the placental barrier. EDC can be found in food, water, and personal care products. They can get into food from the environment and as a result of their migration to food products and cosmetics from packaging. EDCs can disrupt the functioning of the thyroid gland through a number of mechanisms, including disrupting the activation of thyroid receptors and the expression of genes that are related to the metabolism, synthesis, and transport of thyroid hormones (HT). There is a need to strengthen the food safety policy that aimed at the use of appropriate materials in direct contact with food. At the same time, an important action is to reduce the production of all waste and, when possible, use biodegradable packaging, which may contribute to the improvement of the quality of the entire ecosystem and the health of food, thus reducing the risk of developing thyroid diseases.

## 1. Introduction

Exposure to environmental pollution is a global problem that is increasing all the time, according to The Lancet Commission on pollution and health elaboration from 2017. Statistics show that, in 2015–2016, pollution was responsible for around nine million deaths worldwide. The definition developed by the European Union states that environmental pollutants are undesirable materials that have been introduced into the ecosystem through human activities and that endanger human health [1] and cause damage to the ecosystem [2]. Environmental pollution with chemicals, heavy metals, and pesticides leads to environmental degradation, including contributing to soil depletion, air pollution, and groundwater pollution. These substances enter the body through various routes, both respiratory, through the digestive tract and the skin, and may contribute to irreversible damage to health, for example, by their teratogenic and carcinogenic effects. They pose a serious problem in low- and middle-income countries from a public health perspective. Chemicals can enter the environment through fertilizers, including pesticides and herbicides. Pesticides and heavy metals are both persistent pollutants in the environment. There are over 20,000 commercial products available on the global market with a global consumption of 5.2 billion pounds [1,3]. This pollution is mainly exposed to people working on farms, mines, and industrial factories. For heavy metals, the available data also show that around 61 million people in the 49 countries studied are exposed to both heavy metals and other pollutants [1].

**Figure 1:**
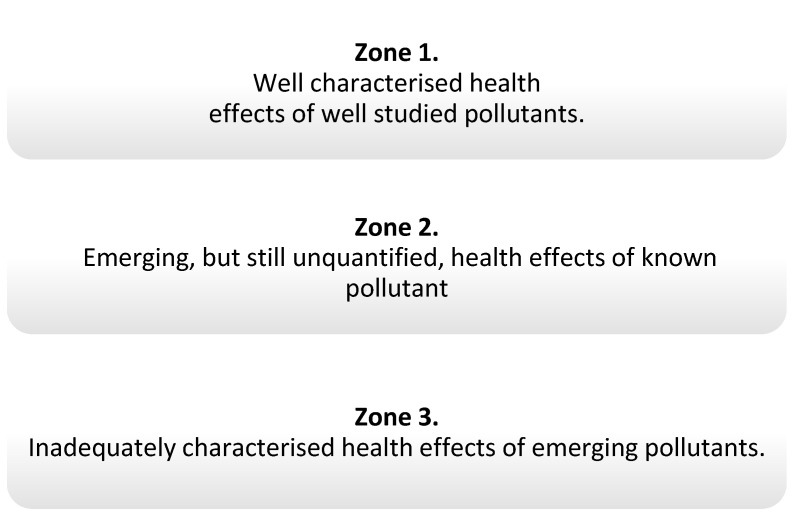
Zones determining the degree of knowledge of pollution according to The Lancet [1].

The Lancet Commission developed some concepts of pollutants and divided them into three zones, defining the level of knowledge about them, depending on the type of pollutants and their health effects (Figure 1). Pollutants from zone 1 are included in the report and, among them, there are endocrine-disrupting chemicals (EDCs) [1], which, through various mechanisms, may interfere with the functioning of hormones [4,5].

**Figure 2:**
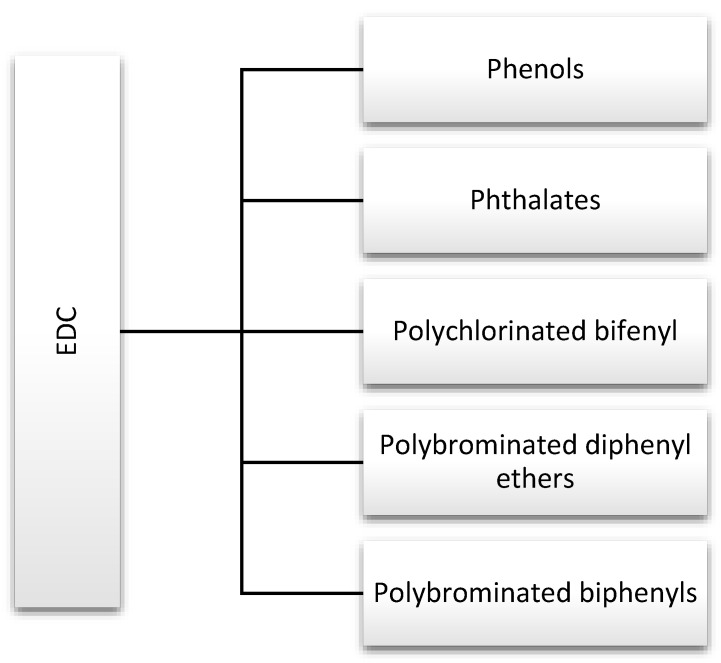
Major endocrine-disrupting chemicals (EDCs) discussed in this review.

Endocrine disruptors, including phthalates, phenols, and flame retardants (FRs), are widespread in the environment. They are produced in large quantities and used in products that are used by many people on a daily basis, including hygiene products, perfumes, food containers, and other plastics [1]. For this reason, they are discussed in this review (Figure 2). Strict rules for controlling exposure to these substances have been introduced due to the large number of scientific reports indicating their negative impact on human health. In addition, actions have been defined to gradually withdraw them from water, the production of industrial chemicals, and plant protection products [6].

Daily exposure to EDC occurs via several routes: diet, inhalation, and direct skin contact [7].

Data show a strong relationship between the EDC and the thyroid gland. These substances affect thyroid hormones (TH) at various levels, but they rarely affect the receptor (TR) itself, as the ligand binding domain is very specific [8]. The most frequently described effects are the effects on the central regulatory system in the hypothalamus and pituitary gland, production and transfer of TH, their function, metabolism, and bioavailability [9,10,11,12,13] (Table 1).

Thyroid diseases are one of the most common chronic diseases [14]. Epidemiological data from 2014 that were presented by the federal agency Statistics Poland (*pol*. *Główny Urząd Statystyczny*—GUS) show that thyroid diseases are one of the most common chronic diseases in women in Poland [15]. In addition, thyroid nodules constitute a serious clinical problem, according to the American Thyroid Association (ATA). It is estimated that nonpalable nodules can be detected in 25% of people aged 19–50 [16,17].

## 2. Phenols and Phthalates

### 2.1. Bisphenol A (BPA)

Bisfenol A (2,2-bis-(p-hydroxyphenyl)-2-propane, BPA) is an organic chemical compound that belongs to the group of phenols. It is widely used in the production of plastics, including polycarbonates and epoxy resins [18]. These materials are used to make protective coatings; ladles are used in food technology [19]. It is also used in the production of varnishes for coating metal products, such as linings for food cans and lids for bottles and water supply pipes; therefore, it can also be found in drinking water [20]. Moreover, heating the pipes may additionally increase its migration [18], therefore hot tap water may be more contaminated by BPA [21]. The substances may also get into the food when heating the cans, plastic packaging, or in the presence of acids and bases, and during storage [18]. Based on this, diet is presumed to be the main source of exposure to BPA, although there is ample evidence that it may also enter the body by differing routes, through dust and air, among others. There are also reports that this substance may pass into the breast milk [20].

Certain dental sealants and composites can also contribute to BPA exposure. It is also found in medical devices, packaging, household cleaning products, as well as cleaning and personal care products [18]. BPA is used in the production of clear and rigid packaging, including food and polycarbonate tableware [19]. This substance is also used in the production of reusable bullets, including baby bottles, although the use of BPA in their production has already been banned in the United States, Canada, and the European Union [19,22,23,24]. Based on current scientific evidence, the European Food Safety Authority (EFSA) panel established a Tolerable Daily Intake of 0.05 mg/kg bw [25].

A large review of 500 peer-reviewed studies by Corrales et al. showed that BPA is widely distributed in the ecosystem. Its actual concentration is significantly higher than the Predicted No Effect Concentration (PNEC) of many countries in Asia, Europe, and North America [26].

#### 2.1.1. Bisphenol A in Food

The research review that was published by Russo et al. presents an analysis of the presence of bisphenols in various food products in 27 European Union countries for six groups of food products from the last five years. It has been shown that BPA can be released from all packaging except glass. They were most often found in soft drinks, energy drinks, cola, beer and juice, and milk-based drinks due to migration from packages [27]. In addition, a study conducted by Bea et al. found that chronic consumption of canned beverages led to increased blood BPA levels [28]. Similar results were obtained with canned vegetables. Moreover, high concentrations of BPA and its analogues are noted in seafood. While higher concentrations are observed in canned fish, pollution of the seas and oceans with municipal and industrial sewage and plasticizers also has a significant impact [27]. A recent study conducted by Barboza et al. investigated the potential relationship between BPA concentration and microplastic contamination of fish. The compound was determined in the muscles and liver of fish: *Dicentrarchus labrax, Scomber colias,* and *Trachurus trachurus*. The lowest BPA concentration in the liver was found (5 ng/gsm) *in T. trachurus* and the highest (302 ng/g dry weight) in *S. colias*, which also had a high concentration (272 ng/g dry weight) bisphenol E (BPE) in the muscles. In addition, the target hazard quation (THQ), hazard index (HI), and estimated daily intake (EDI) of bisphenol were higher than the values that were established by the EFSA [29].

These contaminants are not always the result of the release of compounds from packaging, as migration can occur at any stage of production through contact with utensils and equipment that are used for food processing [27].

BPA is more frequently replaced with analogs of bisphenol F (BPF) (4,4′dihydroxydiphenylmethane) and bisphenol S (BPS) (4,4′-sulfonylbenol) in the production of polycarbonates. They are also used in the production of thermal paper. Although BPA is the most widespread, BPS and BPF are the main food contaminants in the United States. Data from the National Health and Nutrition Examination Survey (NHANES) 2013–2014 showed high levels of BPA, BPS, and BPF in urine samples. However, the median for BPA was the highest in both adults and children [30]. For this reason, it is important to consciously choose products that are BPA-free, do not heat food, and do not store warm food in containers containing BPA (can be marked with the recycling code 3 or 7). It is also worth limiting the consumption of canned food and choosing and storing food in glass containers [20].

#### 2.1.2. Bisphenol A and Thyroid Functions

Bisphenols can affect thyroid dysfunction through a number of mechanisms, including gene expression at the pituitary and thyroid hormone levels and the induction of toxicity of several cell lines. In addition, BPA has been shown to have an antagonistic effect on TR (thyroid receptor), which has an impact on the transcriptional activity and competition with transport proteins [31].

In animal studies, bisphenol A and its analogues have a negative effect on the reproductive system [32,33]. In addition, other data indicate a high influence of BPA on the risk of developing breast cancer [34]. BPA and phthalates are detected not only in urine and breast milk, but also in the amniotic fluid, and, thus, can overcome the placental barrier [35], although not all the studies confirm it [36]. In addition, recent reports indicate a strong relationship between prenatal exposure to BPA and the development of obesity, which indicates its obesogenic properties [37]. BPA may affect adipogenesis through various epigenetic mechanisms, although Longo et al., in their study, found that these changes may be reversible [38]. A study conducted by Derakshsan et al. showed that BPA may also affect thyroid function and deiodinase activity in the early stages of pregnancy (6–14 weeks). BPA was associated with a lower ratio of both FT4/FT3 (free triiodothyronine/free thyroxine) and TT4 /TT3 (total thyroxine/total triiodothyronine), as well as TT4 concentration [39]. On the other hand, a study by Kwon et al. demonstrated lower levels of T3 and T4 in urine with higher BPA exposure than with low exposure with body mass index (BMI) >25.0 kg/m^2^. However, no significant association has been shown for the thyroid-stimulating hormone (TSH), although earlier studies have shown a negative association [40,41]. While Wang et al. showed that the concentration of BPA in urine in the prenatal period was associated with low TSH in overweight mothers, but there was no association with FT4, FT3, and TSH in umbilical cord serum [42]. Moreover, the disturbance of thyroid hormones (TH) levels as a result of prenatal exposure to BPA may be associated with long-term neurobehavioral changes in later age [43]. This may be important, due to the risk of developing subclinical hypothyroidism, as it is believed that leptin plays an important role in obesity-related hypothyrotopinemia and it may increase the development of autoimmune thyroid disease and, consequently, lead to hypothyroidism [44].

BPA can affect the endocrine system in different ways, depending on the degree of exposure, the type of tissue it affects, and gender. BPA as an EDC mimics estrogen by binding to the estrogen receptor, it can activate or inhibit its action, which has been shown in studies in animal models, and its action as an agonist has also been confirmed in humans [45]. A similar effect is observed in the case of its analogues, such as bisphenol S (BPS) and bisphenol F (BPF) [46].

The study conducted by Berto-Júnio et al. investigated the interactions of BPA and BPS with Pax 8 (paired box protein 8) and TTF1 (thyroid transcription factor 1) in sillico on the expression of thyroid genes in an animal model and while using molecular modeling. Pax 8 and TTF1 play an important role in thyroid organogenesis, hormone production, and the maintenance of thyrocyte differentiation. As transcription factors, they regulate the expression of most proteins that are involved in the biosynthesis of thyroid hormones, such as thyroglobulin (Tg), thyroperoxidase (TPO), and sodium iodide symporter (NIS). The study showed that BPS is not a safe alternative to BPA, and it may also affect thyroid disorders [47]. The study of Zhang et al. verified whether bisphenol S (BPS) (4,4′-sulfonylbenol) and bisphenol F (BPF) (4,4′dihydroxydiphenylmethane), like BPA, can disrupt TH signaling through in vitro and in vivo tests. The binding of bisphenol to TR was measured at the molecular level using the competitive fluorescence binding and molecular docking assay. It has been shown that BPS and BPF, like BPA, can interfere with TH signaling. Using the FT3 competitive binding assay, it has been shown that BPS and BPF can bind to both thyroid receptor—α (TRα) and thyroid receptor-β (TRβ). Subsequently, they may exhibit agonist or antagonist activity similar to that of the estrogen receptor. The results of the study confirmed the potential risk of BPS and BPF [48]. The study conducted by Terrien et al. obtained similar results for BPA [49]. The study by Zhang et al. investigated the effect of BPS on the endocrine function of the thyroid gland in zebrafish larvae. Changes in the levels of TH and TSH have been observed by modulating the expression of genes that are related to the hypothalamic–pituitary–thyroid axis (HPT axis), thus leading to BPS toxicity in the thyroid endocrine system. BPS caused a dose-dependent decrease in T4 concentration by 19.5% and 25.7% at exposure to 10 and 30 µg/L, respectively. In the case of TSH, differences were only observed at higher exposure values by 35.6% and 54.6% as compared to the control group. The BPS values in the larvae were similar to those that were observed in food products in the United States, hence the conclusion that they were from strong human exposure to BPS [50].

Andrianou et al., in a pilot case-control study in Cyprus and Romania, examined 212 women for the presence of thyroid nodules. Thyroid nodules >3 mm in diameter were detected in 106 women and 106 healthy women were assigned to the control group. A significant correlation was found between BPA and TSH levels; however, no similar relationship was found for BPF or chlorinated derivatives (ClxBPA). There was also no association between the exposure to bisphenols and FT4 in the serum. The TSH level was lower in the study group than in the control group. However, urinary BPA levels that were adjusted for factors that could affect the outcome (urinary creatinine, disease status, BMI, age, and study site) were higher in the controls than in the nodule group. It could have been influenced by factors, such as diet or the source of exposure [51].

A study conducted by Moriyam et al. showed that BPA may affect the reduced binding of T3 to nuclear receptors and, thus, may also inhibit the transcription process as a result of recruitment of the nuclear hormone receptor corepressor (N-CoR) to TR [52].

The study of Wang et al. investigated the effect of BPA on the volume and structure of the thyroid gland. 718 Chinese children from grades 3–5 of primary school were enrolled in the study. All of the patients underwent anthropometric measurements, ultrasound examinations of the thyroid gland (USG), and urine tests to detect the iodine, creatinine, and BPA levels. The researchers also analyzed the salt they consumed for iodine content. The median urine iodine concentration was 159 μg/L, which was normal for children in this age group (100–199 μg/L) [53,54]. Thyroid volume has been shown to significantly increase with age, BPA, and urine iodine concentration. Children consuming iodized salt had a relatively larger thyroid volume when compared to children consuming non-iodized salt. BPA was detected in 99.9% of urine samples. The median BPA concentration was similar for boys (2.64 μg/g creatinine) and girls (2.35 μg/g creatinine), but it also increased with age (*p* trend = 0.028). Urine BPA was not significantly associated with the risk of goiter, while iodized salt intake was associated with a reduced risk (Odds Ratio (OR): 0.34; 95% CI: 0.14–0.84). Thyroid nodules were carved in 14% of the children. There was an inverse relationship between urinary BPA and thyroid volume and the risk of multiple thyroid nodules in children [54].

Most of the studies showed the effect of BPA on lowering thyroid hormone levels, but it was not always noticeable for TSH values. BPA may also affect the risk of developing thyroid nodules. In addition, there is evidence of a positive relationship between BPA levels and BMI, which might be the cause of subclinical hypothyroidism.

### 2.2. Phthalates (PAE)

Similar to phenols, phthalates (PAE) are an important group of chemical substances that may affect the function of the endocrine [45]. They are salts and esters of phthalic acid, which are used in the production of phthalate-glycerin resins that are used in the production of varnishes, phthalic paints and laminates [55]. They are mostly used in the production of plastics, including polyethylene (PE), polyvinyl chloride (PVC), polyethylene terephthalate (PET), and polyvinyl acetate (PVA) [56]. They are found in many personal care products, including shampoos and lotions. They are also often used as “fragrances” and as plasticizers to change the physical properties of basic plastics, including polyvinyl chloride products, such as shower curtains, floors, as well as packaging and some medical devices [45].

Phthalates are rapidly metabolized and excreted in urine and faeces. Phthalate diester is hydrolyzed to monoester by lipase and esterase in the intestinal epithelium, liver, blood, and other tissues, followed by its systemic distribution [57]. Phthalate metabolites are present in the general population of the US, according to the Fourth National Report on Human Exposure to Environmental Chemicals (Fourth Report). Moreover, significantly higher levels have been found in the urine of women, possibly due to the use of more intensive body care cosmetics [58]. Based on the data that were collected in 2013–2014 as part of the Health and Nutrition Examination Survey (NHANES) cycle, an analysis was made of the diet of chemical exposure of 2212 subjects. It was shown that the consumption of ultra-processed foods (added flavorings, hydrogenated oils, dyes, hydrolyzed protein, emulsifiers) was associated with higher levels of several phthalates, mono- (3-carboxypropyl) (MCPP), mono- (carboxyisoctyl) (MCOP), mono- (carboxyisononyl) (MCNP) and not mono-benzyl (MBzP), ∑DEHP metabolites, or bisphenol. In addition, consuming less processed foods was associated with lower phthalate concentrations [59].

In accordance with Commission Regulation (EU) 2018/2005 of 17 December 2018 Benzyl Butyl Phthalate (BBP), Dibutyl Phthalate (DBP), Diisobutyl Phthalate (DIBP), and Bis (2-ethylhexyl) Phthalate (DEHP), (“four phthalates”) are listed as toxic for reproduction category 1B (“where responses are described following exposure between 3 min. and 1 h and observations up to 14 days”—according to Regulation (EC) No. 1272/2008 of the European Parliament and of the Council of 16 December 2008) [60]. According to a 2008 European Union report, there is a need to reduce the risk of bis (2-ethylhexyl) phthalate (DEHP) use due to its negative health effects, including problems with the testes, fertility, and kidney toxicity [61].

**Figure 3:**
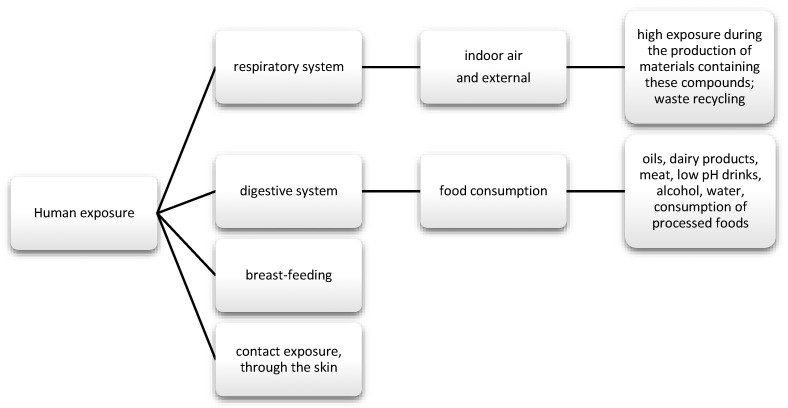
Main routes of exposure to phenols and phthalates.

The data show that as much as 50% of DEHP is absorbed through the gastrointestinal tract; in the case of diethyl phthalate (DEP), it is about 80%. Nevertheless, there are reports suggesting that phthalates may also enter the body through inhalation (Figure 3). Regarding exposure by workers producing polyvinyl chloride (PVC) products, DEPH exposure can account for as much as 46.7% of the daily dose taken for highly exposed groups. In addition, exposure may also occur via skin contact [62]. Studies also frequently detect phthalate metabolites, mainly monobutyl phthalate (mBP), mono-ethylhexyl phthalate (mEHP), and mono-isononyl phthalate (mNP) in breast milk [63].

#### 2.2.1. Phthalates in Food

PEA can get into food by leaching and migrating from unit packaging, including lids to glass containers, food foil, aluminum, and cardboard packaging (obtained from recycling) [64]. The review conducted by Giuliani et al. analyzed articles on the presence of PAE in food: oils, meat, dairy products, and plants, as well as beverages, such as soft drinks, alcoholic beverages, and water. For alcoholic beverages, contamination has been shown to mainly occur during the production phase. Additionally, there were no significant differences in impurities, depending on the packaging. The most contaminated were commercial wines, both white and red. In the case of drinking water, the highest amounts of PEA were observed in still water, which was stored at higher temperatures of 22–60 °C and in a capacity of 0.5 l (high surface area to volume ratio). Non-alcoholic drinks with potassium sorbate are more susceptible to contamination than water. Moreover, the greater the acidity of the product (pH = 3), the greater the migration of PAE. PAE and their metabolites have also been found in coffee and tea. They came from plastic packaging and tea bags with a plastic liner (bags with filter paper). The dependence of the concentration of pollutants on the time of exposure was observed. Because of PAE’s affinity for fat, it can be found in oils and fat-rich foods. As in the case of wine production, it was observed that contamination of oils can mainly occur at the production stage, during the harvest of the raw materials, but, in this case, it also increased in the subsequent stages of production. It has also been shown that physical refining can completely remove the phthalates. Similarly, for dairy and meat products, the higher the fat content, the higher the PAE concentration in the product. The stage of processing milk into dairy products exerted the greatest influence on the content [65]. No significant concentration of DEPH in meat was found in a study by Tsai et al. in the Taiwanese population, specifically in samples of unpackaged pork and chicken. Nevertheless, the authors of the study suggest a phthalate monitoring program for health effects [66]. On the other hand, in the case of dairy products, or rather milk, PEA can get into the milking stage, which is used in the pipes used during milking and in transport from the dairy. DEHP was the most frequently described pollutant. Therefore, some countries have banned the use of DEHP in milk tubes. The migration of contaminants may also occur during storage in cartons that are lined on the inside with foil used for food products [63]. In the case of plant products, the cultivation method, fertilizers, and pesticides used are important. Plants that were grown in greenhouses using plastic foils were more polluted than in open fields [65].

#### 2.2.2. Phthalates and Thyroid Function

In a study by Wang et al. that was conducted in China, employees who dealt with the recycling of plastics had higher levels of urinary phthalates, TT3, and T3 /T4 when compared to the control group. However, these relationships were non-monotonic, therefore further research is needed to explain the relationship between thyroid disorders and phthalates [67]. Phthalates are rapidly metabolized, and then excreted in the urine and partly in the faeces. They have an estimated short half-life and are quickly excreted from the body [68].

Based on the 2019 systematic review, which included 29 publications, a positive relationship between phthalates and adiposity measures was found [69]. Moreover, DEHP, like BPA, may influence the development of thyroid disorders, including the development of autoimmune thyroid disease [70]. A cross-sectional study by Souter et al. has shown that the urinary concentration of DEHP metabolite mixtures may be a major factor in reducing FT4 and FT3, as well as TT4 and TT3, and may therefore affect thyroid function or regulation of the HPT axis. However, no association with autoimmunity was found in women suffering from infertility [71]. A study that was conducted in Taiwan by Tsai et al. showed that children and adolescents <18 years of age are exposed to high DEHP exposure. More than half of the 250 participants were exposed to DEHP contaminated food, which exceeds the European Food Safety Authority’s Recommended Tolerated Daily Intake of DEHP (<50 µg/ kg/d). However, in this study, no association was found between substance consumption and thyroid function [72].

There is evidence that the effects of these substances can affect thyroid hormone levels and may be gender-dependent. In cross-sectional study of the United States (U.S.) using the data from the National Health and Nutrition and Examination Survey 2007–2008 population, a significant negative correlation with total T4 in men was shown, which may be closely related to the influence of these substances on hormone synthesis, while, in women, it was associated with higher T3 levels. The different effects of these substances may be related to the frequency of exposure due to the fact that women generally use certain cosmetics more often, such as sunscreen [73]. Another study evaluating the gender differences in phthalene exposure in children over three years of age found that there was an inverse relationship between the levels of metabolites other than DEHP (MnBP, MiBP, MBzP, and MEP) and FT4 in girls, which was not observed for boys. However, this relationship was not statistically significant [74].

A study conducted by Liu et al. investigated the effects of phthalates in oncogenesis, including the occurrence of thyroid cancer and benign nodule in Wuhan, China. Some phthalates, such as urinary monomethyl phthalate (MMP), mono (2-ethyl-5-hydroxyhexyl) phthalate (MEHHP), and mono (2-ethylhexyl) phthalate (MEHP), have been shown to be associated with the development of thyroid cancer and nodule [75]. A study by Huang et al. that was conducted in Taiwan assessed the exposure of phthalates to thyroid function. 11 metabolites measured by liquid chromatography with tandem mass spectrometry were used for the analysis. Serum thyroxine levels, free T4, T3, thyroid stimulating hormone, and thyroxine binding globulin (TBG) were analyzed for assessing thyroid function. Growth hormone homeostasis was measured as levels of insulin-like growth factor 1 (IGF-1) and insulin-like growth factor binding protein 3 (IGFBP3). The study showed that exposure to phthalates affects thyroid function. FT4 was negatively correlated with urinary MEPH levels (β = −0.013, *p* = 0.042) and MEHHP (β = −0.030, *p* = 0.003), while it was positively correlated with urinary MMP (β = 0.014, *p* = 0.037) after adjusting the factors that may interfere with the results, such as age, BMI, gender, urinary creatinine levels, and TBG. In addition, there was a positive association between urinary MEHP levels and IGF-1 levels, which means phthalenes may also affect growth hormone homeostasis [76]. Wu et al. showed that preschool exposure to phthalates can affect HT and growth. The study found that children in urban areas are more exposed to the effects of mono-phthalate metabolites (MPAEs). Additionally, most of the MPAEs were positively associated with FT3 and FT4. On the other hand, the concentration of IGF-1 decreased by 0.082 ng/mL and 0.132 ng/m, respectively, with an increase in monomethyl phthalate (mMMP) and mono-n-butyl phthalate by 1 ng/mL in both cases [77].

Di- (2-ethylhexyl) phthalate (DEHP) is also widely used in the production of building materials, clothing, cosmetics, cleaning, and personal care products. A study conducted by Kim et al. showed, both in vivo and in vitro, that DEHP induces cell proliferation and damages DNA. It turns out that DEHP can induce DNA damage and affect cell proliferation, even at low micromolar ranges; in addition, in vivo data confirm that DEHP can affect thyroid tissues at doses from 0.3 mg/kg [78].

Most of the observations showed a negative association of phthalates with T4 levels, especially in women, which may be the result of more frequent use of cosmetics than men. The participation of phthalates in oncogenesis and occurrence of thyroid nodules has been reported, as in the case of phenols.

## 3. Flame Retardants (FRs)

Organo-bromine compounds are widely used chemicals in industrial production and they are added to many products to reduce their flammability. They are used in the automotive industry and electrical equipment. They are also utilized in the production of plastics, textiles, and polyuteratene foams. The use of certain agents is currently restricted or prohibited in the European Union. However, like the other compounds that were discussed in this article, they have the ability to accumulate in the environment [79,80,81]. Among them, we can distinguish polychlorinated biphenyls (PCBs), polybrominated diphenyl ethers (PBDE), and polybrominated biphenyls (PBB) [80]. New FR compounds are still being developed, which still require research due to the limited data on their toxicity and occurrence [82].

### 3.1. Polychlorinated Biphenyls (PCB)

**Figure 4:**
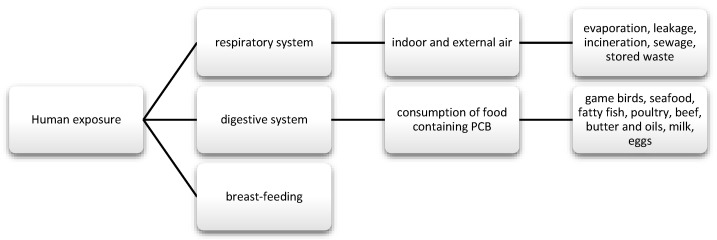
Main routes of exposure to polychlorinated biphenyls (PCBs).

PCBs are a group of chemical compounds derived from biphenol. Biphenol consists of two benzene rings that are connected by a carbon-carbon bond in the 1′1 position with different number of chlorine atoms [83]. PCBs can be metabolized into hydroxylated polychlorinated biphenyls (OH-PCB) and methylsulfonyl-PCB (MeSO2-PCB). Except that MeSO2-PCBs are characterized by low reactivity, lipophilicity, and bioaccumulation [84]. Until now, 209 have been known and described and 180 of them were present in commercial mixtures under names such as: Fenclor (Italy), Phenoclor (France), Clophen (Germany), Sanoterm (UK), Kanechlor (Japan), Sovol (Russia), and Delor (Czech Republic) Aroclor (USA) [83], and about 130 are identified in environmental samples of congeners, although they are not naturally present. As mentioned, PCBs are lipophilic compounds [85] and their lipid solubility increases with the number of chlorine atoms in the molecule [86]. Therefore, they bioaccumulate in food chains. In addition, PCBs are stable and resistant to physical, chemical, and biological degradation, due to which they are able to persist in both the environment and human body [85]. Their half-life in the environment is 10 to 15 years [87]. These substances were often used in capacitors and transformers due to their physicochemical properties, stability and non-flammability. They also provide good eclectic insulation [88,89]. Moreover, they were used as plasticizers for paints, inks, glues, paper, and lubricating oils [88]. Their metabolism depends on the number and location of chlorine in the molecule and has an influence on toxicity. The smaller the number of chlorine atoms, the faster their metabolism [90]. Human exposure and the toxic effects of PCBs occurs most often via the oral route along with the diet, and then they pass from the intestinal contents into the systemic circulation. Nevertheless, there are strict regulations regarding maximum levels and harmful PCBs that are similar to dioxins in food production [91]. The EFSA panel agreed that the daily intake of this nutrient should not be around 2 pg/kg bw [21]. In addition, the substances may be absorbed through the skin, as well as through the respiratory tract and breast milk (Figure 4) [92,93].

It has been proven that PCBs, at the highest levels, are detected in more developed, industrialized countries. The highest concentration in human milk samples was recorded in the Czech Republic and Slovakia, the lowest, among others, in Sweden, Italy, and Poland [94]. In Poland, the daily intake of PCBs, together with mother’s milk, is below the tolerated daily intake [95].

In 1979, the US Congress banned PCB production [96]. In Europe, on the basis of the Stockholm Convention on Persistent Organic Pollutants, which was signed in 2001, the removal and disposal of PCB-containing materials is to be initiated by 2025. In Poland, the convention was ratified in 2008 [97]. Like other substances of this type, they have a long half-life in the environment [98], so it can be assumed that they will persist in the environment for many more years.

#### 3.1.1. Polychlorinated Biphenyls in Food

Data that were published by EFSA on the risk to human and animal health show that the main sources of exposure to PCBs are butter, cheese, fatty fish, especially farmed trout and salmon, and household meat [82]. Chen et al. investigated the presence of polybrominated diphenyl ethers PBDE and PCBs in eight samples of semi-skimmed milk and non-fat, which is used in California. The mentioned compounds were detected in all of the tested samples. In the case of PCBs, the most frequently detected congers were: PCB-118, PCB-101, and PCB-128. However, the concentration was below the tolerated value that was determined by the FDA [99]. A study in Taiwan investigated, among others, the presence of PCBs in various food samples from different parts of the country. The highest levels were found in fish, followed by seafood, crabs, as well as large and sea fish. In smaller amounts in eggs and dairy products, and the smallest in cereals, vegetables, and fruit. The differences in concentrations strictly depended on the location from which a given product came from [100]. However, in a study by Arrebola et al., with 1880 inhabitants of Spain, despite the close correlation of PCB levels with fish and dairy products, a relationship was also indicated for the consumption of raw fruit and, in the case of some congers, also with the consumption of vegetables. However, this could be due to the more frequent consumption of these products in southern countries. Therefore, the differences in exposure of other people are largely due to the diet used, and they are also based on the place of residence [101]. At this point, it is worth mentioning a study that was conducted in the Silesian Voivodeship in Poland. This area is characterized by a relatively high concentration of PCBs, but also other doxins, such as polychlorinated dibenzodioxins (PCDD) and polychlorinated dibenzofurans (PCDF). This study compared samples of food that was sourced directly from the farm to those that can be bought in-store. Almost all of the samples of eggs and fresh meat from farms showed high concentrations of all toxins, exceeding the maximum levels [102].

#### 3.1.2. Polychlorinated Biphenyls and Thyroid Functions

The study conducted by Gaum et al. showed both cross-sectionally and longitudinally negative correlations between PCB and FT3. Four-year exposure to PCBs resulted in a reduction in FT4 and an increase in TSHRab (antibodies against receptors TSH) [103]. In the study, Curtis et al. demonstrated that higher levels of PCBs are associated with higher levels of FT4 and a FT3 higher ratio: FT4. The data also indicate a special risk of developing thyroid disorders in people exposed to the compounds in childhood and even in the prenatal period [104]. Based on data from the Janus Serum Bank cohort, Lerro et al. conducted a nested case-control study on the incidence of thyroid cancer in the Norwegian population. Of the 36 congeners, there was an inverse relationship between thyroid cancer for only one PCB congener (PBC-114) and stronger associations among the youngest birth cohort for three others (138/158 and 153). Age may probably be an important determinant of risk and exposure early in life may increase the risk of thyroid cancer [105]. However, a study of 743 people living in the vicinity of a former PCB manufacturing facility found no association between exposure to the substances and thyroid hormones, TSH, and other indicators of thyroid dysfunction. Only an inverse relationship was observed between the PCB and TT3 levels, although it was not clinically significant. There were also no differences in the number of PCBs and gender [106]. Likewise, the study by Zani et al. showed no effect of PCBs on the occurrence of endocrine and metabolic diseases, despite the long-term persistence of compounds in the serum [107]. In the case of hydroxylated polychlorinated biphenyls (OH-PCB) toxicity, a study by Itoh et al. demonstrated that maternal exposure during pregnancy may increase the maternal and neonatal FT4 levels, although they were still within the normal range [108], although not all of the previous studies confirmed this relationship [109]. On the other hand, the increased level of TSH may result from an increase in T4 in the negative feedback system [108]. In a study involving women in the first trimester of pregnancy and newborns, a relationship was only found between the concentrations of some OH-PCB isomers and neonatal TSH [110]. This, in turn, can lead to an overactive thyroid gland in the mother and, thus, increases the risk of miscarriage, premature birth, and low birth weight [108]. The increase in FT4 levels may be due to the fact that some hydroxylated compounds can inhibit the activity of D1 that is responsible for the conversion of T4 to T3 [109]. However, there are still many disparate results on the effects of PCBs and OH-PCB on the TSH and FT4 levels in humans. It turns out that PCB can increase and decrease T4. In contrast, both in the case of PCBs and OH-PCBs, it was observed that exposure of a pregnant woman to the action of these substances may affect the TSH and T4 values in the newborn.

PCBs disrupt the thyroid gland at the DNA level, affecting transcription. They also exert an influence on proteins that are related to the sodium-iodine symporter (NIS), thyroid peroxidase (TPO), and thyroglobulin (Tg). In addition, it can reduce the expression of mRNA, deiodinase 2 (D2), and deiodinase 3 (D3) [111]. The study by Soechitram et al. also confirmed the negative effect of PCBs on D3 activity involving 100 mother-newborn pairs. PCB showed a positive correlation with T3/rt3 in umbilical cord serum, but no correlation with TSH was found [112]. Similar results were obtained in a study conducted by Dirnick et al. involving 180 people who were recruited from the Department of Endocrinology of the Antwerp University Hospital between 2009–2012. All of the subjects were diagnosed with obesity or type 2 diabetes, but they were euthyroid. PBC and their metabolites were associated with lower FT4 levels, without a concomitant increase in TSH [113]. In a large study that used data from 4998 children and adolescents (seven to 17 years of age) and 2501 adults (1071 men and 1430 women aged 20–75 years), the interrelationship between several endogenous and exogenous effects and thyroid volume and function was investigated. Increased levels of fT4 have been reported in people living in highly polluted areas [114]. The variation in T4 seems to depend on the level of exposure. It may be related to the action of PCBs on deiodotyrosine, which are responsible for catalyzing the deiodination reaction of thyroid hormones. On the other hand, changes in the level of thyroid hormones may result from the action of PCBs on deiodotyrosines, which are responsible for catalyzing the deiodination reaction of thyroid hormones.

### 3.2. Polybrominated Diphenyl Ethers (PBDE)

PBDEs are another group of compounds that belong to flame retardants. They are formed due to the bromination of diphenyl ether. There are 209 PBDEs congeners. Additionally, in this case, many studies confirm their high affinity to fats, which means that they can easily accumulate in all organisms [81,115].

**Figure 5:**
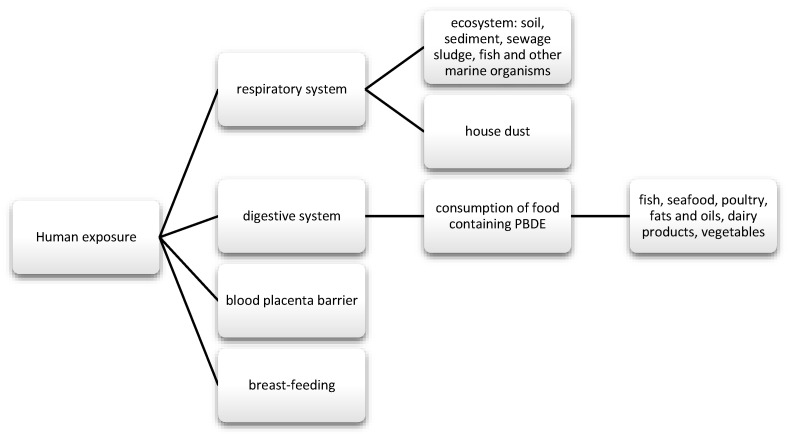
Main routes of exposure to PBDE.

They were commonly used for the production of foams for upholstered furniture, fabrics, and other plastics [45]. There are three types of commercial mixtures: pentabromodiphenyl ether (pentaBDE), octabromodiphenyl ether (octaBDE), and decabromodiphenyl ether (decaBDE) [116]. These compounds can be released during the recycling process and during their exploitation [117]. The content of ether—terabromodiphenyl and pentabromodiphenyl (components of pentaBDE) as well as hexabromodiphenyl and heptabromodiphenyl (components of octaBDE)—must not exceed 0.001% by weight of the product, if it is present in substances, flame-retardant preparations, or articles, according to the European Commission Directive 757/2010 of August 2010 (10). Where the products concerned are manufactured wholly or partly from recycled or derived materials from waste prepared for reuse, they may constitute up to 0.1% by weight of the product content [118]. They are used as flame retardants, also in wire insulation and cars [119]. They get into the human body through the respiratory and digestive tract, and they also cross the blood-placenta barrier (Figure 5) [120,121,122,123].

#### 3.2.1. Polybrominated Diphenyl Ethers in Food

Brominated flame retardants (BFRs) getting into the food chain can contaminate food mainly of animal origin, which is why they are found in meat, fish, seafood, and dairy products [80,124]; they can also be found in plants, including legumes and grain products [112] or potatoes [125]. In addition, research indicates a higher content of new BFRs in greenhouse plants, which is probably due to the longer growth time of vegetables grown in this way. The analysis of individual plant parts confirmed the different absorption potential of BFRs by greenhouse and conventionally grown plants. Moreover, a higher concentration of compounds was observed in cucumbers than in tomatoes, which the authors of the study associate with the volume of the product. Sun et al. pointed out the need to change the way of growing plants in greenhouses, the construction of which consists of plastic. In turn, from crops in arable lands, the lowest concentration was found for rice that was grown on wet soils (paddy soils) [126]. E-waste recycling areas are particularly exposed to pollution. The research review by Sci et al. took data from various areas of China and their pollution levels into account, including the two countries of Guiyu and Taizhou. In countries where global waste is received, it has been shown that tri to hepta BDE levels from these areas are significantly higher than in others, and the consumption of contaminated food in Guiyu was 55,889 ng/d, on average per kilogram of body weight it was 931 ng, which was half the chronic reference dose for penta-BDE established by EPA, namely 2000 ng/kg bw/day. From the food products, high concentrations of BRFs have been reported in freshwater fish, which indicates the highly concentrated water in these areas [124]. The presence of BFRs in fish and, more precisely, PBDEs, has also been confirmed in other studies [124,127,128]. In the case of seafood, it is suspected that some of these compounds may reduce the bioavailability after processing, but this has not been demonstrated for frying and cooking eel. Nevertheless, the abundance of BRFs from seafood is considered to be relatively high [128].

#### 3.2.2. Polybrominated Diphenyl Ethers and Thyroid Functions

Endocrine disorders may occur as a result of exposure of the organism to PBDEs. Because of the similar structure of PBDEn decay compounds to thyroid hormones, they may affect its function as a result of morphological and histological changes [129]. They are likely to interact with steroid hormone receptors and suppress the normal function of thyroid hormones, which may also contribute to neurological abnormalities in the developing fetus. Many observational studies have demonstrated the presence of PBDEs in maternal urine [45]. The study by Hansen et al. investigated the effect of three phthalate diesters: di-n-butyl phthalate (DnBP), diethyl phthalate, di- (2-ethylhexyl) phthalate (DEHP) and two monoesters (mono- (2-ethylhexyl) phthalate (MEHP), mono-n-butyl phthalate) on the function of primary thyroid epithelial cells. DEHP and its monoester, MEHP, had an inhibitory effect on the secretion of cyclic adenosine 3′-5′-monophosphate from cells. Additionally, MEHP also influenced the secretion of thyroglobulin (Tg) from cells. None of the analyzed diesters showed an effect on the expression of thyroid-specific genes (TSHR, Tg, NIS, TPO). The analysis showed that human thyroid cells were able to metabolize phthalates, and no effect on hormone secretion was observed in an in vitro study [130].

In a study conducted by Liu et al. in an animal model, an association was shown between plasma TH and the level of thyroid-related gene transcription in three tissues (in the liver, thyroid, and brain). They were related to liver FR concentrations. Hepatic PBDE levels were negatively correlated with plasma TT4 and TT3. In addition, the plasma TH levels and hepatic PBDE concentrations were correlated with the transcription of genes engaged in metabolism (type of deiodinases—1, 2,and 3) and synthesis (thyroglobulin and a sodium iodide symporter) in the thyroid gland [131]. Similar results were obtained in a study with a North American adult cohort. Exposure to PBDE is associated with a decrease in serum T4 levels [132,133], and it may also reduce the binding of T4 to serum binding proteins [132]. Hydroxylated PBDE (OH-PBDE) can be converted to sulfates of polybrominated biphenyls, which, in turn, can disrupt the TH system by binding to transporter TH or TR proteins [134]. In people with thyroid cancer, OH-PBDEs are associated with a decreased FT4 value and an increase in TSH [135]. However, in the case of individual congeners, these relationships may be completely independent or even opposite to the concentrations of circulating thyroid hormones. It is possible that they have competing mechanisms of toxicity with each other [136]. A study conducted by Guo et al. showed that increased exposure to PCBs, PBDEs, and new FRs can reduce TSH, thyroxin-binding globulin (TBG), TRa expression, but increase D1 expression. Chemicals alter some TH regulatory proteins. In addition, they disrupt gene expression and secretion, and then transport and degradation, and they affect TH receptors Other studies also confirm that D1 may be involved in the biotransformation of other BDE-209 congeners [137,138].

Thyroid cancer is one of the most common cancers of the endocrine system and its incidence is constantly increasing. Additionally, studies show that women are three times more likely to develop thyroid cancer than men [139]. In a case-control study, Deziel et al. investigated the relationship between serum PBDE concentrations and the risk of papillary thyroid cancer (PTC) in women that were exposed to single or combined exposure to multiple PBDE congeners. 462 women aged 21–84, Caucasian, participated in the study. There was no evidence of an increased risk of developing thyroid cancer in relation to exposure to PBDE, although a positive relationship was found between the concentration of BDE-209 in house dust and PTC [140], similarly to the work of Hoffman et al. In this study, BDE-209 was associated with smaller and less aggressive thyroid nodules [141]. A recent case-control study involving US military personnel confirmed that elevated levels, in the case of BDE-28 congener, may also be associated with an increased risk of PTC. The association has been observed in tumors >10 mm and it was stronger in women [142].

A Canadian cross-sectional study analyzed the prevalence rates for hypothyroidism in 745 women aged 30–79 years. Brominated diphenyl ethers (BDEs) levels have been shown to be associated with increased morbidity, especially in the 51–79 years old group [143]. Another study investigated the effect of pentaBDE (DE-71) on human cells in vitro, more specifically the production of cyclin adenosine monophosphate (cAMP) and Tg in the culture medium by a competitive radioimmunoassay and enzyme immunoassay, respectively. Additionally, quantitative real-time PCR analysis of the thyroid specific genes was performed. DE-71 showed an inhibitory effect on the functions of thyroid cells, including Tg release from thyrocytes. At a dose of 50 mg, Tg production decreased by 71.9% and cAMP by 95.1% as compared to control. Moreover, the expressions of mRNA encoding Tg, TSH, and TPO were significantly inhibited [144].

It is observed that people with a higher content of it or with metabolic syndromes are much more exposed to the negative effects of PBDE due to the possibility of accumulation of compounds in adipose tissue, thus they have a much lower FT3 [145]. There is considerable evidence that they disrupt hormone transport and thyroid function [146]. Exposure to BDE congeners (47, 99, 100) is closely related to the occurrence of thyroid disease, especially in postmenopausal women, possibly due to altered estrogen levels during this period [146,147]. The effect of decabromodiphenyl ether (BDE-209) and decabromodiphenyl ethane (DBDPE) exposure on the HPT axis on an animal model in the study by Wang et al. was investigated, which had not been tested before. It was observed that a high dose of BDE-209 (500 mg/kg) significantly lowered the TT3, TT4, FT3, and FT4 levels and increased TSH and TRH. In the case of DBDPE, the 28-day exposure only resulted in a significant decrease in FT3 in the 50 and 500 mg kg bw/day dose groups. As with BDE-209, an increase in TSH and TRH levels was also observed with high dose DBDPE exposure. Hypothyroidism may develop as a result of these changes in the body. Moreover, the histopathological analysis of the thyroid gland showed structural changes in the gland in the studied groups due to oxidative stress. There was a significant decrease in the dose-dependent superoxide dismutase activity in both cases and in glutathione in the group exposed to BDE-209 at a dose of 500 mg/kg bw/day. Significant increases in malondialdehyde were also observed, especially after BDE-209 [148]. However, in men, there is also an association between exposure to PBDE and decreased TSH levels [145,149]. Although there is still insufficient evidence to support a positive relationship between exposure to PBDEs and risk of thyroid cancer, exposure to PBE may nevertheless cause a reduced release and transport of thyroid hormones, thus leading to the development of hypothyroidism.

Studies involving pregnant women and newborns confirm that exposure to PBDE, especially BDE-29 and 47, is related to the concentration of T4 and T3 with increasing exposure, in both cases [150]. Some of the data indicate that the already low prenatal exposure to PBDE may be associated with behavioral disturbances in children and a decreased concentration of HT in umbilical cord plasma [151]. Children with high prenatal and infant BDE exposure have lower TSH levels when compared to children that were exposed to lower exposure early in life [152]. Some data suggest that exposure to PBDE may be associated with reduced head circumference in an infant, but larger trails are still needed [153]. Children are more likely to have lower T4 and higher FT3 levels as the exposure increases. Childhood exposure to PBDEs subclinically disrupts the function of thyroid hormones, which may result in the development of hypothyroidism [154]. In studies on animal models, the effect of PBDEE on thyroid function has also been confirmed. An effect on circulating HT concentrations was observed as a result of exposure of the offspring during the prenatal period. Offspring from mothers that were exposed to BDE-99 showed decreased survival, body length, and an increase in malformations [155]. In some cases, only the female offspring were affected [156]. PBDE has also been proven to influence prenatal exposure to disturbances in the functioning of the thyroid gland, as in the previously discussed PCB.

### 3.3. Polybrominated Biphenyls (PBB)

Polybrominated biphenyls are brominated PCB analogs. There are three groups of PBB compounds: octabromobiphenyls, hexabromobiphenyls, and decabromobiphenyls [157]. They consist of two aromatic rings and bromine atoms. Their numbering corresponds to that of PCB and PBDE. PBBs are also characterized by low water solubility (and this ability decreases with increasing bromination), bioaccumulation, and persistence in the environment [157,158]. On the other hand, they show the ability to dissolve in fats [157]. Many years of observations prove that high contamination with PBB agents can persist in the environment for many decades and, thus, constantly affect organisms. Some evidence suggests that chronic exposure is closely associated with thyroid disease, especially in women [159]. PBBs are added to plastics, such as televisions, computer monitors, and plastic foam textiles [160].

Since the contamination of PBB food in Michigan in 1973, when the greatest chemical disaster took place, in which harmful compounds were mistakenly entered into animal feed, restrictions on the use of substances in industry have been introduced [161]. Until the problem was identified, local residents were at a high risk of consuming food containing PBB. Observational studies have reported many cases of breast cancer, gastrointestinal cancer, and fatalities among local residents [162]. The maximum concentration of PBB in homogeneous materials may be 0.1%, according to Directive 2011/65/EU of the European Parliament and of the Council [163].

**Figure 6:**
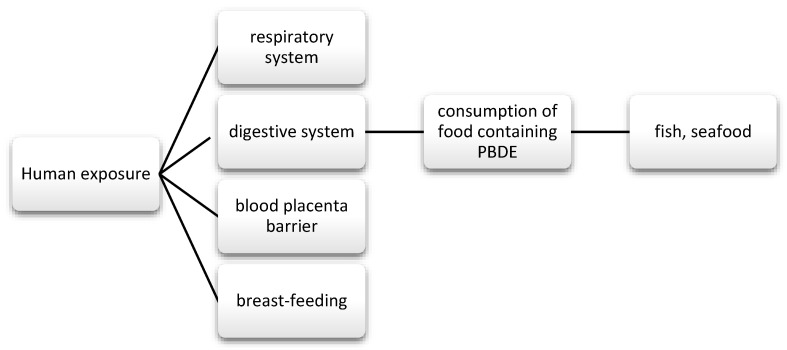
Main routes of exposure to PBDE.

Research conducted from 2012–2015, i.e., approximately 40 years after the accident in Michagan, showed that people who were directly exposed to chemicals are still significantly burdened with higher concentrations when compared to the general U.S. population; this also applied to people who were born after the accident. This may confirm the effect of prenatal exposure, of their passage through the placenta, and with maternal milk. Studies have confirmed that exposure to PBB can occur via different routes, both via the gastrointestinal tract, as well as inhalation and skin absorption (Figure 6) [164].

#### 3.3.1. Polybrominated Biphenyls in Food

Food tests for PBB mainly include the analysis of products, such as fish and seafood. A study by Falandysz et al. from the University of Gdańsk showed that products from the Baltic Sea are characterized by an increasingly lower content of PBB than in the years of 1972–1993, when the pollution level was similar to the North Atlantic Region. One compound that was detected was PBB-77 (dioxin-like PBB) in both dietary supplements (cod liver oil) and food [161]. Another study examining the content of PBB in fish from the Baltic Sea and other fish available for purchase at a market in Poland showed that the average concentration of these substances in Baltic fish was higher than in carp, but also lower than in fish from the North Sea. Additionally, the highest levels of PBB were observed in the Baltic salmon, slope, and sea bream, and relatively low in herring. For the same species, the PBB concentration was the higher the higher the fat content. This study also confirmed that PBB may accumulate more in the liver than in the tissues. Pork, beef, and butter samples were also analyzed, but none of them contained PBB above the LOD (limit of detection) [165]. Nevertheless, the United Kingdom (UK) analysis from 2006 did not show that shellfish consumption was associated with significant exposure to PBB. The daily consumption was at the tolerated level. Basically, oysters and mussels contained more contaminants than scallops [166]. It turns out that the PBB levels can decrease in food due to the heat treatment. The study conducted by Zabik et al. showed that spray drying reduces PBB in whole and skim milk. It has also been observed that cooking poultry under pressure reduces the content of these substances in the thighs, meat, and skin by 36% [167]. This can be a useful observation in the case of food products with a higher content of these substances.

#### 3.3.2. Polybrominated Biphenyls and Thyroid Functions

The chemical structure of PBDE and PBB congeners is structurally similar to thyroid hormones; therefore, it is believed that their hydroxylated metabolites can compete with HT transport proteins and receptors. [168] PBBs may impact on HPA axis function, although dates described it are limited. It can lead to problems with the production and release of corticosterone, thus affecting the feedback mechanisms [12]. It is possible that hydroxylated PBB (OH-PBB) may interfere with the function of thyroid hormones by bonding to the ligand binding domain of the thyroid receptor β (TRβ) [13]. The study by Guo et al. examined the level of exposure of children at the age of 10 on PBDE and other new FRs (chlorinated and brominated flame retardants). Non-monotonic relationships between PBDE, FRs, TH, and TSH were observed. Serum levels of PBDE + FRs were positively associated with T3 levels due to the dominant component of BDE-209 [169]. In people exposed to PBB in childhood or in the prenatal period, the presence of the compound in serum is associated with higher FT3, a higher ratio of FT3:FT4, and lower T4. In addition, it has been shown that people exposed to higher exposure before 16 years of age have a lower FT4 and a higher FT3 [104]. In a case-control study, Deziel et al. investigated the relationship between serum PBB concentrations and the risk of papillary thyroid cancer (PTC) in women. There was a positive association between brominated biphenyl 153 (BB 153) exposure and PTC risk [140]. Most of the research that was conducted on PBB concerned people living in Michigan and its vicinity. A study conducted by Jacobson et al. also found an association of PBB-153 with thyroid diseases, mainly with hypothyroidism. The incidence was higher in women who have been exposed to PBB in the past [154]. Similarly, in the study by Yard et al., the assessment of the occurrence of thyroid diseases showed that women exposed to PBB are more often diagnosed with thyroid diseases (13.9%) than men (2.6%). When compared to the control group, women with thyroid disease had an increased risk of being overweight or obese [170]. In the case of PBB, the relationship between prenatal exposure and disturbances in the functioning of the thyroid glands is also confirmed. It can possibly lead to hypothyroidism or PTC. This is especially true in high-exposure populations.

## 4. Conclusions

Both phenols, phthalates and substances belonging to flame retardants, polychlorinated biphenyls, polybrominated biphenyls, and polybrominated diphenyl ethers are compounds that are widely distributed in the environment. Endocrine disrupting chemicals can disrupt the function of the thyroid gland by influencing the hypothalamic–pituitary–thyroid axis, gene expression, and competing with thyroid hormone transporters. In addition, many data indicate their relationship with obesity and metabolic syndrome, which may additionally affect changes in the hormone levels, thus leading to the development of subclinical hypothyroidism and autoimmune thyroid disease. In studies, the effect of these substances on the reduction of thyroid hormones and the presence of thyroid nodules was observed, especially in women.

Although many restrictions and regulations limiting their use have been introduced in recent years, they are still detected at various concentrations in humans, in animal organisms, and plants. The high-risk group includes people living in industrial areas of e-recycling waste, where the consumption of these substances, along with contaminated food, may exceed the acceptable standards. These compounds can be found in foods that are consumed on a daily basis. Particular attention should be paid to those that are packed in plastic packaging, films, and canned food, which can be lined from the inside with a layer of polymer containing, among others, bisphenol A. For this reason, it is also worth choosing food grown in a traditional, conventional way or in greenhouses that are made of safe materials.

In addition, phenols and phthalates are also found in many personal care products, including lotions, shampoos, and one of the main routes of human exposure is through skin contact. For this reason, the content of phenols and phthalates in cosmetics should be constantly monitored to ensure human safety. One solution would be the use of natural and organic packaging on a larger scale by producers. T the use of glass packaging by consumers may also be an alternative, as there are increasing points where you can fill your containers with selected cosmetics and cleaning agents.

It seems important to strengthen the food safety policy aimed at the use of appropriate materials that come into direct contact with food due to the high problem of environmental pollution around the world. At the same time, an important action is to reduce the production of waste, which significantly contributes to the deterioration of the quality of the entire ecosystem and the health quality of food, thereby increasing the risk of developing thyroid diseases.

## Figures and Tables

**Table 1:** Mode of action EDCs in in the hypothalamus–pituitary–thyroid axis [9,10,11,12,13].

Groups of EDCs	Mode of Action
BPA (bisphenol A) and other phenols	Interference with the activation of thyroid receptors (TR): α1 and β1; Inhibition of the transcriptional activity of genes; Influence on the expression of the TR gene in cells of the thyroid gland and pituitary gland;
PAE (phthalates)	Expression of genes related to: - metabolism, - synthesis, - transport of thyroid hormones; Downregulation TRH receptor (thyroid-releasing hormone) in the hypothalamus; Downregulation in thyroid-stimulating hormone (TSH) receptor the thyroid gland; Upregulation of TRH protein and mRNA (messenger ribonucleic acid) levels in the pituitary gland;
PCB (polychlorinated bifenyl)	Cytochrome P45 (CYP1A1) induction through aryl hydrocarbon receptor (AhR) activation—influence on the TH receptor;
PBDB (polybrominated diphenyl ethers)	Binding to the TR; Inhibition of triiodotyrosine (T3); Binding of transporters (TRR and thyroid binding globulin (TBG); Activity of thyroid deiodinase (D); The metabolism of thyroid hormones;
PBB (polybrominated biphenyls)	Competition with thyroid hormone (TH) transport proteins; Bonding to the ligand binding domain of the TRβ Inhibition of TH synthesis;

## Data Availability

Not applicable.
